# New-Onset Nephrotic Syndrome in a Child Associated With COVID-19 Infection

**DOI:** 10.3389/fped.2020.00471

**Published:** 2020-08-20

**Authors:** Siddharth A. Shah, Helen P. Carter

**Affiliations:** ^1^Department of Pediatrics, University of Louisville, Louisville, KY, United States; ^2^Bowling Green Internal Medicine and Pediatric Associates, Bowling Green, KY, United States

**Keywords:** case report, new-onset, nephrotic, swelling, COVID-19, pediatric

## Abstract

**Introduction:** The COVID-19 outbreak has become a worldwide public health emergency. The renal histopathological features of acute tubular necrosis or thrombotic microangiopathy have been previously reported in adults with severe COVID-19 infections. In children, the renal manifestations associated with COVID-19 disease are not widely reported. Here we describe a case report of a child with new-onset nephrotic syndrome associated with COVID-19 infection.

**Case Presentation:** An 8-year-old boy with no previous significant medical history presented with bilateral eyelid and facial swelling soon after his parents were diagnosed with COVID-19 infection. He had diarrhea but no fever or shortness of breath. At 1 week after the onset of swelling, the boy tested positive for the COVID-19 virus. Based on clinical findings of significant proteinuria (urine protein and creatinine ratio of 11.4), hypoalbuminemia (serum albumin of 2 g/dl), and hypercholesterolemia (total cholesterol of 384 mg/dl), he was diagnosed with nephrotic syndrome. He responded well to standard-dose prednisone treatment for nephrotic syndrome. At 1 week after starting the prednisone treatment, he went into clinical remission. Lymphopenia continued to be present for 4 weeks after the onset of symptoms. There were no complications related to clot formation or secondary infections with this presentation.

**Conclusion:** COVID-19 can be associated with new-onset nephrotic syndrome in children. The patient responded well to the standard-dose prednisone treatment that is typically used for new-onset nephrotic syndrome.

**Summary:** We describe the unique presentation of COVID-19 in a child as new-onset nephrotic syndrome. We offer insight on the success of standard treatment of nephrotic syndrome with COVID-19.

## Introduction

The novel coronavirus or COVID-19 outbreak started in China in December 2019 (1). The first case of COVID-19 in the United States was confirmed on January 20, 2020 (2). Subsequently, the outbreak has become a worldwide pandemic and a global public health emergency (3). In general, children are reported to have a less severe presentation and better prognosis compared to adults with COVID-19 infection (4). In adults, acute kidney injury and the need for dialysis are commonly seen in critical patients with COVID-19 infection (5). The novel coronavirus acts through angiotensin-converting enzyme 2 (ACE2) on body cells. Animal studies have shown a high expression of ACE2 in tubular epithelial cells and podocytes in kidneys (6). A report that summarized the renal pathological findings on 26 postmortem kidney specimens from China showed the presence of COVID-19 virus particles in tubular epithelial cells and podocytes as detected by electron microscopy (7). The involvement of podocytes in the glomerular filtration barrier by the virus may lead to proteinuria. A prospective cohort study of 701 patients in China hospitalized with COVID-19 infection showed that proteinuria was present in 43.9% of patients, although acute kidney injury was reported in only 5.1% of patients (8). In this report, proteinuria, along with acute kidney injury, was associated with increased mortality in hospitalized patients (8). Will the possible podocyte involvement by the novel coronavirus alter the presentation or treatment course of COVID-19-associated nephrotic syndrome? We could not find previous case reports of new-onset nephrotic syndrome in children associated with COVID-19 infection. We describe the following report.

## Patient Information

We report a case of an 8-year-old boy who presented with swelling on the face and eyelids in April 2020. Both of his parents were diagnosed with COVID-19 infection on the day prior to his initial presentation of eyelid swelling. His mother presented with symptoms of chills, cough, and shortness of breath, and his father presented with symptoms of fever and congestion, leading to the diagnosis of COVID-19 infection.

## Clinical Findings

The boy never developed a fever, but he had gastrointestinal symptoms of vomiting and diarrhea in association with facial swelling at presentation. There was no reported breathing difficulty. The facial swelling was initially thought to be related to allergies, and he was treated with anti-allergic medication without response. The swelling progressed, and he also developed scrotal edema and +2 pedal edema. At 1 week after the initial presentation, he was evaluated at his pediatrician's office. He displayed a weight gain of 3.3 kg (from 28 to 31.3 kg) since the onset of swelling. The blood pressure measured was in the normal range for his age and height. He tested positive for COVID-19 infection using nucleic acid amplification testing on a nasopharyngeal swab specimen.

He did not have a significant past medical history and had a normal birth history. There was no family history of kidney disease.

## Diagnostic Assessment

The urinalysis showed +4 protein and +2 blood with a specific gravity of 1.036. The random urine protein and creatinine ratio was high at 11.4. The comprehensive metabolic panel showed acceptable serum electrolytes with serum sodium of 141 meq/L, serum potassium of 4.8 meq/L, and serum tCO_2_ of 23 meq/L. Renal function was satisfactory as assessed by blood urea nitrogen (BUN) of 14 mg/dl and serum creatinine of 0.32 mg/dl. The serum albumin was significantly low at 2 g/dl. The transaminases were normal with AST of 16 IU/L and ALT of 29 IU/L. The lipid panel showed that total cholesterol was elevated at 384 mg/dl, LDL cholesterol was elevated at 272 mg/dl, and triglyceride level was at 127 mg/dl. The complete blood count showed a white blood cell count of 14.1 × 10^3^/ul, hemoglobin of 14.7 gm/dl, and platelet count at 468 × 10^3^/ul. The lymphocyte count was low at 6.3%, while the neutrophil count was 88.4%. The complement C3 was normal at 148 mg/dl, and the complement C4 was normal at 54 mg/dl. The patient was clinically diagnosed with nephrotic syndrome based on nephrotic range proteinuria, significant hypoalbuminemia, edema, and hypercholesterolemia. We were not sure if the new-onset nephrotic syndrome was related to abnormal immune response secondary to COVID-19 infection or there was a random association.

## Therapeutic Intervention

The typical first-line treatment of new-onset nephrotic syndrome in children is steroids, but in this case, we were concerned about immune system suppression with steroid use because of the reported high virulence associated with COVID-19 infection. Additionally, the effects of COVID-19 on organs such as kidneys were still under investigation. However, our patient had a progressive increase in edema, including the presence of scrotal edema. Our risk assessment for thrombotic complications with both COVID-19 and the active nephrotic syndrome was high. We did not anticipate appropriate immune response to COVID-19 in the active phase of the nephrotic syndrome because of the expected loss of immunoglobulins in the urine. We discussed the benefits and the risks of using steroids in this situation with the family. After introspection, we decided to start steroid treatment for our patient to achieve nephrotic syndrome disease remission. He was started on prednisone at the dose of 30 mg, twice a day, as per standard nephrotic syndrome treatment guidelines. He was also started on a proton pump inhibitor for gastrointestinal protection and counseled on a low-sodium diet.

## Follow-Up and Outcomes

At 2 days after starting prednisone, the patient experienced an increase in testicular swelling. His weight had increased by 1 kg. The edema was treated with metolazone (diuretic), given for 3 days. At 5 days into treatment with prednisone, the protein level on a urine dipstick showed an improvement. At 7 days into treatment, he was in remission, with urine protein and creatinine ratio in the normal range at 0.2. The urine output had improved, and his weight had trended down to 28 kg. The complete blood count that was repeated at 1 week after the treatment of steroids still showed a low lymphocyte count of 9.4%. There were no signs or symptoms of secondary infections or clots during his initial treatment with prednisone. While the patient was in remission, we obtained serological and coagulation studies to evaluate his immune response to COVID-19 and the persistence of thrombotic risk factors. The COVID-19 IgG ELISA antibody test was positive. The plasma D-dimer was <0.27 mg/L (normal range: <0.5 mg/L), the antithrombin III function was mildly below average at 76% (reference range: 83–128%), and the fibrinogen level was average at 333 mg/dl (reference range: 200–400 mg/dl). We did not administer thromboprophylaxis. The lymphopenia continued to be persistent, with a value of 10% at 4 weeks after treatment with steroids. The patient was continuing to receive standard-dose prednisone treatment for new-onset nephrotic syndrome at the time of writing this report. He will be monitored closely, and his clinical course will be studied over time.

## Discussion

The novel coronavirus or COVID-19 is reported to be associated with renal pathologies, including acute tubular necrosis, (7) thrombotic microangiopathy, (9) and collapsing glomerulopathy (10). To our knowledge, this was the first case report of new-onset nephrotic syndrome reported in a pediatric patient associated with COVID-19 infection at the time of submission. Although the burden of disease from COVID-19 is less severe in children, they may present with illnesses related to an abnormal immune response to COVID-19 infection. There are reports of pediatric multisystem inflammatory disorders similar to Kawasaki disease in children with COVID-19 infection (11). Childhood nephrotic syndrome is one of the most common pediatric immune-mediated renal disease (12). New-onset nephrotic syndrome has been reported previously with other viral outbreaks, such as H1N1 (13).

The boy in our case report was likely infected with COVID-19 through family contacts. He did not have the typical symptoms of COVID-19 infection, such as fever or shortness of breath. He did have gastrointestinal symptoms, which are a less common symptom of COVID-19. There was no reported anosmia. Lymphopenia has been reported previously in pediatric patients as associated with COVID-19 infection, (4) but the presence of lymphopenia at 4 weeks after the onset of symptoms was peculiar for this patient. Although the edema was progressive, the proteinuria and swelling improved quickly with standard-dose prednisone treatment. The patient also had microscopic hematuria. However, given the normal renal function as evaluated by serum creatinine level, normal BUN levels, normal blood pressure, and normal serum complements, a proliferative glomerular disorder was not high on differentials at presentation, and renal biopsy was not performed. The response to treatment of this nephrotic syndrome presentation was similar to that for minimal change disease.

This report provides one perspective on the treatment course and the outcome on the new-onset nephrotic syndrome in children in association with COVID-19 infection. More studies are needed to understand the benefits and the risks of steroid treatment in this condition. Our patient had a positive IgG antibody response to COVID-19. It may still be helpful to study if children with a more severe and prolonged phase of active nephrotic syndrome and COVID-19 infection can generate an appropriate serological response. The lack of renal biopsy-derived histopathological analysis is the major limitation of this report. The association between COVID-19 and new-onset nephrotic syndrome could also be just random.

## Conclusion

COVID-19 infection may be associated with new-onset nephrotic syndrome in children. Although the clinical presentation of new-onset nephrotic syndrome associated with COVID-19 infection is not significantly different from nephrotic syndrome associated with other viral infections, further case studies are needed to confirm this finding. The new-onset nephrotic syndrome related to COVID-19 may respond well to standard-dose prednisone treatment, but more studies are needed to assess the benefits and the risks of steroid treatment in this condition.

## Patient's Perspective

(Note from the patient's mother)

“Having this virus myself was so terrifying, but to see what it has caused my child has been much worse. Watching him change so drastically to the point I don't even recognize my own child has been devastating. From gaining significant weight from fluid and steroid treatments, it has not only affected how we see him, but it has really affected his self-esteem.”

## Data Availability Statement

All datasets presented in this study are included in the article/supplementary material.

## Ethics Statement

This case report was reviewed and approved by the local institutional review board. Written informed consent was obtained from minor's legal guardian (parent-mother) for the publication of any potentially identifiable images or data included in this article.

## Author Contributions

SS Pediatric nephrologist involved in the initial care of this patient with nephrotic syndrome. HC Primary Pediatrician involved in the care of this patient with nephrotic syndrome. We collaborated on the care of this patient together. All authors contributed to the article and approved the submitted version.

## Conflict of Interest

The authors declare that the research was conducted in the absence of any commercial or financial relationships that could be construed as a potential conflict of interest.
